# Introduction of the DUNDRUM Triage Urgency Tool to a Medium Secure Unit in Bed Crisis

**DOI:** 10.1192/bjo.2024.559

**Published:** 2024-08-01

**Authors:** Leanne Duthie, Alistair Morris

**Affiliations:** NHS Lothian, Edinburgh, United Kingdom

## Abstract

**Aims:**

At a time of increased pressures across the whole forensic estate, The Orchard Clinic Medium Secure Unit faced the additional challenge of having to close multiple acute admission beds.

This led to challenges in determining how to triage patients in the face of multiple external pressures, frustrations for clinicians managing severely ill patients in prison and human rights concerns for those unfit to stand trial but remanded to custody to await a bed.

The need for an objective tool to aid triage decisions became apparent. We therefore piloted the use of the DUNDRUM triage urgency manual, a structured professional judgement tool to aid triage decisions for forensic units.

The aims of introducing this tool were to ensure decisions are more consistent and reliable, ensure scientifically valid items are not forgotten, make decision making processes more transparent, demonstrate equality of access to services and reduce chance of serious error.

**Methods:**

This audit reviewed all acute admissions to The Orchard Clinic between Aug 22–Aug 23. This covered a period 6 months prior to the introduction of the tool and 6 months after.

In order to determine if the use of the tool improved our triage making decisions the Dundrum score was retrospectively calculated for admissions and those on the waiting list during the first 6 month period of the audit. The same information was recorded for those following the introduction of the tool in the second 6-month period.

**Results:**

Prior to introduction of the DUNDRUM, the team's triage decisions were not in line with validated tools, those with lower DUNDRUM scores were prioritised over those with higher scores. Following introduction of the tool our triage decisions improved. Common themes emerged when we analysed the reasons why our triage decisions were out of line with validated tools. These included patients in hospital settings taking precedence over those in prison, patients admitted without prior discussion at bed management meetings, legal urgency taking precedence over clinical and lack of available HDU space.

**Conclusion:**

Prior to the introduction of the DUNDRUM triage urgency manual the audit demonstrates that the team's triage decisions were not in line with validated tools. This improved following training and use of the tool at bed management meetings. The Orchard Clinic has now formalised use of this tool within bed management meetings. We are currently in the process of re-auditing over a 12-month period.

